# The accuracy of object motion perception during locomotion

**DOI:** 10.3389/fpsyg.2022.1068454

**Published:** 2023-01-12

**Authors:** Oliver W. Layton, Melissa S. Parade, Brett R. Fajen

**Affiliations:** ^1^Department of Cognitive Science, Rensselaer Polytechnic Institute, Troy, NY, United States; ^2^Department of Computer Science, Colby College, Waterville, ME, United States

**Keywords:** optic flow, moving objects, navigation, flow parsing, self-motion, motion

## Abstract

Human observers are capable of perceiving the motion of moving objects relative to the stationary world, even while undergoing self-motion. Perceiving world-relative object motion is complicated because the local optical motion of objects is influenced by both observer and object motion, and reflects object motion in observer coordinates. It has been proposed that observers recover world-relative object motion using global optic flow to factor out the influence of self-motion. However, object-motion judgments during simulated self-motion are biased, as if the visual system cannot completely compensate for the influence of self-motion. Recently, Xie et al. demonstrated that humans are capable of accurately judging world-relative object motion when self-motion is real, actively generated by walking, and accompanied by optic flow. However, the conditions used in that study differ from those found in the real world in that the moving object was a small dot with negligible optical expansion that moved at a fixed speed in retinal (rather than world) coordinates and was only visible for 500 ms. The present study investigated the accuracy of object motion perception under more ecologically valid conditions. Subjects judged the trajectory of an object that moved through a virtual environment viewed through a head-mounted display. Judgments exhibited bias in the case of simulated self-motion but were accurate when self-motion was real, actively generated, and accompanied by optic flow. The findings are largely consistent with the conclusions of Xie et al. and demonstrate that observers are capable of accurately perceiving world-relative object motion under ecologically valid conditions.

## Introduction

Success in many common locomotor tasks requires that we accurately perceive the motion of objects that move independently from us. When the observer is stationary, the motion of an object can be perceived based on the object’s local optical motion, since it reflects the object’s movement through the world (Harris and Drga, 2005; Gray et al., 2006; Duke and Rushton, 2012). Oftentimes, however, the observer also moves, and the visual system confronts the added complication that the observer’s self-motion may influence the object’s optical speed and direction.

Object motion in this case could be described with respect to a reference frame that either moves with the observer (*observer-relative;* depends on self-motion) or remains fixed with respect to the stationary world (*world-relative;* independent of self-motion). The frame of reference upon which humans rely to perceive object motion has important consequences in tasks that depend on the observer’s action capabilities, such as target interception and obstacle avoidance (Fajen, 2013). Consider the scenario wherein an observer must decide whether to pass in front or behind a moving obstacle, such as a pedestrian crossing the observer’s future path. To successfully perform the task, the observer must account not only for obstacle’s motion, but also the minimum and maximum self-motion speeds that are maintainable. Relying on the retinal pattern of motion to coordinate self-motion would be problematic because different combinations of self-motion and object motion result in the same optical object motion. On the other hand, the world-relative motion of the object is by definition independent of the observer’s self-motion. It has therefore been argued that humans may perceive object motion in a world reference frame (Fajen, 2013; Fajen et al., 2013). This could explain why manipulations of global optic flow (which simulates self-motion) influence judgments of the object’s trajectory (Smeets and Brenner, 1994; Brenner et al., 1996; Regan and Gray, 2000; Fajen and Warren, 2004; Gray et al., 2006; Fajen and Matthis, 2013). In addition to its role in the control of locomotion, the ability of moving observers to perceive the motion of other objects in a world-relative reference frame could play a role in a variety of perceptual functions. The present study investigates object motion perception in one such ecologically relevant situation where observers actively generate self-motion through locomotion.

**Figure 1:**
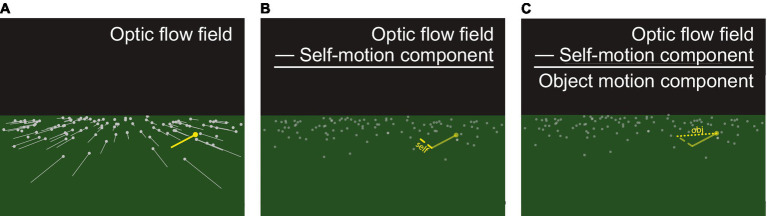
**(A)** The forward movement of an observer over a ground plane generates a radial optic flow field (gray). In the moving reference frame of the observer, the motion corresponding to an object (yellow) reflects the sum of the observer’s self-motion and the independent movement of the object. To recover the world-relative object motion (“obj” in **C**), the visual system could subtract the self-motion from the optic flow field (“self” in **B**).

To recover the world-relative motion of an object during locomotion, the visual system could factor out the component of the object’s local optical motion that is due to the observer’s self-motion, a process that has been termed *flow parsing* (Rushton and Warren, 2005). This follows because the optic flow field represents the sum of the observer’s self-motion and the object’s independent movement, and subtracting out the observer’s self-motion component leaves the motion due to the object’s movement through the world (Figure 1). The global optic flow field is a powerful source of information about the observer’s speed and direction of self-motion and may be used by the visual system to estimate the observer’s self-motion for flow parsing. Support for flow parsing comes from a series of experiments in which human observers view displays that simulate self-motion and make judgments about the direction of an independently moving object. (Self-motion is said to be “simulated” in these experiments in the sense that the stimuli simulate the visual experience of moving through the environment, but the observer is not actually moving.) The general finding is that direction judgments more closely correspond the object’s movement through the world rather than the pattern of motion on the screen (Rushton and Warren, 2005; Warren and Rushton, 2007; Matsumiya and Ando, 2009; Warren and Rushton, 2009).

Although these results are consistent with flow parsing, a frequent finding in studies of visual flow parsing is that the judged direction of object motion falls in between the observer-relative and world-relative directions (Matsumiya and Ando, 2009; Dokka et al., 2013; Dupin and Wexler, 2013; Niehorster, 2013; Niehorster and Li, 2017). This suggests that the visual system may not completely factor out the observer’s self-motion, at least under the conditions used in these studies. The discrepancy has been quantified using a gain factor that defines the degree to which the visual system discounts the observer’s self-motion to arrive at human object motion judgments. Humans judge the direction of object motion within displays that simulate self-motion with gains of around 0.5, where a gain of 1 implies that the visual system fully discounts the observer’s self-motion (Dupin and Wexler, 2013; Dokka et al., 2015). Gains similar to those produced by humans emerge from a neural model proposed by Layton and Fajen (2016) that recovers object motion from optic flow. This study demonstrates that visual flow parsing could involve interactions between brain areas MT and MSTd, which are known to be involved in motion perception (Layton and Fajen, 2016). The transformation from observer (retinal) to world reference frames in the model relies on feedback signals from MSTd neurons that respond to the global optic flow field (e.g., radial expansion). The availability of binocular disparity (Layton and Fajen, 2020) and monocular depth information in the scene (Layton and Niehorster, 2019) modulates the activation of model MSTd and thereby may yield an incomplete recovery of world-relative object motion through weaker feedback signals.

Incomplete flow parsing has also been reported in studies involving real rather than simulated self-motion. In two of the conditions in Dokka et al. (2013), for example, subjects were passively translated on a moving platform with or without visual self-motion information to test the contribution of vestibular input. Judgements of object motion were more accurate when optic flow and vestibular signals provided congruent information about self-motion, but flow parsing gains were below 1.0 (95% CI = 0.53–0.62). Such findings indicate that visual and vestibular self-motion information are not sufficient for complete flow parsing. However, because self-motion was passive, it remains possible that flow parsing is accurate during actively generated self-motion.

Indeed, Xie et al. (2020) found that subjects could accurately estimate world-relative object motion when self-motion was real rather than simulated, actively generated by moving one’s own body through the world, and accompanied by visual self-motion information. Their study was conducted in an ambulatory virtual environment that was viewed through a head-mounted display (HMD). Subjects judged whether a moving object approached or receded over the ground surface under three conditions: (1) the Non-visual condition, in which self-motion was real and actively generated by walking in the absence of optic flow, (2) the Visual condition, in which self-motion was simulated by optic flow while the subject remained stationary, and (3) the Combined condition, in which self-motion was real, actively generated, and accompanied by congruent optic flow. As in previous studies, they found that when either visual (e.g., optic flow) or non-visual (e.g., vestibular, somatosensory, proprioceptive) information is available, but the other is absent (i.e., in the non-visual and visual conditions), humans factor out less than 100% of the self-motion. However, flow parsing was accurate and flow parsing gains were not significantly different from 1.0 when both visual and non-visual self-motion information was available.

The findings of Xie et al. (2020) are important because they demonstrate that observers are capable of accurately perceiving world-relative object motion during self-motion, provided that self-motion is actively generated and accompanied by optic flow. Nevertheless, to extract a quantitative estimate of the flow-parsing gain, it was necessary for Xie et al. to exercise precise control over stimulus properties and strictly limit stimulus duration. For example, the moving object was a small dot that occupied 1 deg. of visual angle and had negligible optical expansion. The dot moved at a speed that was fixed in retinal coordinates, which means that its speed in the real world varied with observer motion. Furthermore, the object was only visible for 500 ms. Subjects were instructed to look at the fixation point at the beginning of each trial until the moving object appeared. If we assume that it took at least 150 ms to shift gaze from the fixation point to the moving object (Krauzlis et al., 1999), subjects were only able to visually track the moving object for at most 350 ms.

This approach offers the advantage of allowing for precise control over properties of the stimulus. However, the conditions were quite unlike those that are encountered in the real world, where objects optically expand, move at speeds that do not depend on observer motion, and persist for longer durations. The present study takes a complementary approach, sacrificing some control over stimulus properties and forgoing the estimation of flow parsing gain so that the accuracy of flow parsing could be assessed under more ecologically valid conditions. The conditions used in the present study differed from those in Xie et al. (2020) in several respects. First, the moving object was a 0.5 m high cylinder with a 0.1 m radius rather than a small dot with negligible optical expansion. Second, the object moved over the ground plane at a speed that was fixed in world coordinates rather than retinal coordinates. Third, the object was visible for 2 s rather than 500 ms. Fourth, the ground surface was densely textured and there were vertical posts to provide rich motion parallax. Lastly, fixation was not required and subjects could move their eyes freely. The overall pattern of results was strikingly similar to that reported in Xie et al. providing converging evidence for the conclusion that accurate flow parsing is possible.

**Figure 2:**
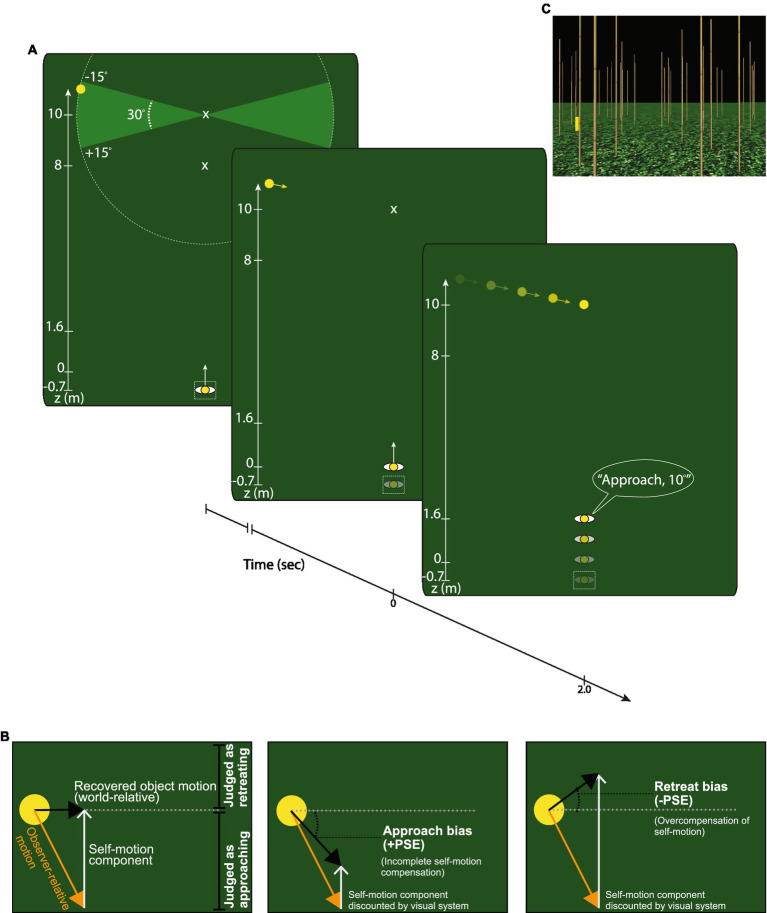
Overview of Experiment 1. **(A)** Top view of the experiment. A cylindrical object appeared in the distance to one side of the central axis, defined by the direction that the subject was instructed to face. The object moved along a trajectory within ±15° of the horizontal and vanished upon reaching the central axis. During each trial, the subject walked (Real Walking condition), stood still (Stationary condition), or viewed simulated self-motion (Simulated self-motion condition). Subjects verbally reported whether the object appeared to approach, retreat, or move at a perpendicular angle relative to their locomotor axis. If the object appeared to approach or retreat, they also judged the trajectory angle. **(B)** Conditions: The three panels depict how perceived object motion in world coordinates would be affected if the visual system accurately compensates (left panel), undercompensates (middle panel), and overcompensates (right panel) for self-motion. A tendency to judge horizontal trajectories as approaching (middle panel), as indicated by a positive point of subjective equality (PSE), would be consistent with the visual system incompletely compensating for self-motion. A tendency to judge horizontal trajectories as retreating (right panel; negative PSE) would be consistent with the visual system overcompensating for self-motion. **(C)** Scene view: Screenshot of the textured virtual environment.

The present study reports the results of two experiments designed to assess the accuracy of object motion perception during self-motion. In Experiment 1, subjects made judgments about the trajectory of a moving object (Figure 2A) while they either walked (Real Walking condition), stood still (Stationary condition), or viewed simulated self-motion (Simulated self-motion condition). By comparing judgments in the Real Walking and Simulated self-motion conditions, we could assess whether object motion perception is indeed more accurate when self-motion is real and actively generated. Experiment 2 replicated Experiment 1 using a different judgment task and included a new condition to determine the accuracy of object motion perception based on non-visual self-motion information alone.

## Experiment 1

In each of the three conditions of Experiment 1 (Real Walking, stationary, and simulated), subjects verbally reported whether the object appeared to approach, retreat, or move at a perpendicular angle with respect to the path of locomotion. If they perceived that the object was approaching or retreating, they also estimated the angle of approach or retreat. Verbal judgments were analyzed to extract measures of the point of subjective equality (i.e., angle at which the object trajectory was judged as neither approaching nor retreating) and discriminability between approaching and retreating trajectories. Thus, the main independent variables were self-motion condition (Real Walking, Stationary, or Simulated) and object trajectory and the main dependent variables were PSE and discriminability.

The Stationary condition serves as a control to assess object motion estimates without self-motion. If the active generation of self-motion (e.g., walking) plays an important role in flow parsing, then object trajectory angles should be perceived more accurately in the Real Walking condition than in the Simulated self-motion condition. Given the similarity between the Simulated self-motion condition and the conditions of previous studies on visual flow parsing (Matsumiya and Ando, 2009; Warren and Rushton, 2009; Xie et al., 2020), we expect subjects to exhibit a bias toward judging objects as approaching, consistent with the visual system discounting less than 100% of the observer’s self-motion (Figure 2B, center panel).

The experimental design allows for testing of differences across conditions in terms of the discriminability of object trajectory angle judgments. Dokka and colleagues found that discriminability was reduced when self-motion was real compared to when it was simulated – that is, the improvement in flow parsing gain was accompanied by a drop-off in discriminability (Dokka et al., 2013). On the other hand, Xie et al. (2020) found no significant differences in discriminability between these two conditions.

### Methods

#### Ethics statement

The experimental protocol was approved by the Institutional Review Board at Rensselaer Polytechnic Institute and in compliance with the Declaration of Helsinki. All subjects gave informed consent in writing before participating in the experiment.

#### Participants

Fourteen subjects between the age of 18 and 21 years (M = 18.7 years) who were enrolled in undergraduate psychology courses participated in the experiment and were awarded extra credit. All subjects reported having normal or corrected-to-normal vision. We planned to collect data from 12 subjects, but two additional subjects signed up. We did not want to exclude their data so we ended up with two additional subjects.

#### Equipment

The experiment was conducted in a 6.5 × 9 m ambulatory virtual environment laboratory. Participants wore a nVis nVisor SX111 stereoscopic HMD with a resolution of 1,280 × 1,024 pixels per eye and a diagonal field of view of 111°. There was approximately 40° of horizontal overlap between the two eyes. An Intersense IS-900 motion tracking system recorded the position and orientation of each subject’s head. Data from the tracking system were used to update the position and orientation of the viewpoint. The virtual environment was created using the Vizard Virtual Reality Toolkit 3.0.

#### Virtual environment

Figure 2A depicts a plan view of the virtual environment. Trials began at the start location where participants stood in a narrow 0.4 × 0.4 × 2 m translucent box and faced a distant alignment marker above the horizon, parallel to the z-axis. The alignment marker disappeared when the trial began.

The virtual environment consisted of a large planar ground surface with a grassy texture and a dark sky (Figure 2C). An array of bamboo-textured vertical posts that attached to the ground surface at various positions in depth enhanced the motion parallax experienced by subjects during self-motion. Posts were distributed by defining a grid of 1.8 × 1.8 m squares and randomly placing one post in each square in a position that varied from trial to trial.

Subjects made judgments about the trajectory of a short (0.5 m height × 0.1 m radius) yellow moving cylinder that glided over the ground plane. The cylinder initially appeared at some position along a circular arc with a radius of 5 m centered on the observer’s central axis 8 or 10 m from the origin (see Figure 2A). The object glided along the ground surface for 2 s toward the central axis at one of the following angles: 0°, ±1°, ±2°, ±3°, ±5°, ±7°, ±9°, ±12°, ±15°. Positive (negative) angles indicate retreating (approaching) trajectories (Figure 2A). The initial position of the object was set so that it would arrive at the central axis at depths of 8 m (Near) or 10 m (Far) relative to the origin after moving along its 5 m path.

#### Data analysis

We performed all data analysis in MATLAB R2022a.

#### Procedure

To prepare for each trial, subjects stood in a designated home location within the virtual environment, facing the bamboo posts. The start of each trial was indicated by the sound of a whistle and the appearance of the object (initially stationary) on either side of the central axis. In the Real Walking condition, subjects were instructed to begin walking forward upon hearing the whistle and maintain a normal speed. In the Simulated self-motion condition, simulated self-motion at a speed of 0.8 m/s commenced 0.5 s after the whistle. We chose 0.80 m/s because this was the mean walking speed that was exhibited by subjects in the Real Walking condition during pilot testing. Mean walking speed in the Real Walking condition in the actual experiment was 0.83 m/s. We also measured subjects’ position relative to the midline when the cylinder stopped moving to verify that there was no systematic veering. The mean distance from the midline was 0.0016 m. Thus, the self-motion in the Simulated self-motion condition closely matched the mean walking speed and direction in the Real Walking condition. In both conditions, the object started to move once subjects reached a distance of 0.7 m from the starting position. Self-motion and object motion continued until the object reached the central axis, at which point it disappeared and subjects verbally reported whether the object appeared to approach, retreat, or move at a perpendicular angle (0°) with respect to their path of locomotion (see Figure 2A). If the object appeared to approach or retreat, subjects also judged the object’s trajectory angle relative to the axis perpendicular to their path of locomotion. Subjects were instructed to judge how the object was moving relative to the stationary environment rather than relative to their position (which changed as they moved). The only motion in the Stationary condition occurred due to the object’s movement and any small head movements made by the subject. The lack of virtual displacement of the observer in the Stationary condition would have caused a discrepancy in the relative depth of the object compared to the other conditions. To better equate the final relative depths, we shifted the object trajectories in the Stationary condition toward the observer by 1.6 m, equivalent to the distance that subjects traveled by moving for the 2 s at 0.8 m/s. This changed the initial object depth to 6.4 m in the Near condition and 8.4 m in the Far condition.

One to three days prior to, and again immediately before the main experiment, subjects completed four practice blocks to acclimate to the virtual environment, gain confidence with moving around while wearing the HMD, and become familiar with the task and the instructions. Feedback about the accuracy of verbal judgments was provided during practice but only in the Stationary condition. This was done to help subjects calibrate to the virtual environment. No feedback was provided during the other practice blocks or during the actual experiment.

The main experiment consisted of three blocks, corresponding to each of the self-motion conditions. Subjects completed the Stationary condition second, between the Real Walking and Simulated self-motion conditions, which were counterbalanced. In each block, we manipulated the object’s trajectory angle (17 trajectories within ±15°), final object depth relative to the origin (8 m and 10 m in the Real Walking and Simulated self-motion conditions and 6.4 and 8.4 m in the Stationary condition), and starting side (left to right). Each 0° trajectory appeared twice within a block, resulting in 72 trials in total.

#### Psychometric analysis

As will be explained in Results and Discussion, we performed a psychometric analysis on the sign of the angular judgments (i.e., angles judged as approaching or retreating). For each trajectory angle condition, we calculated the proportion of trials on which the object was judged as approaching, excluding from the denominator trials with cross judgments (19.84% of all trials) so as not to introduce bias. We then fit the following sigmoid to each subject’s mean data in the Real Walking, Stationary, and Simulated self-motion conditions using least-squares (MATLAB function *lsqcurvefit*):


Eq. 1 fθ;s,p=11+e−sθ−p

**Figure 3:**
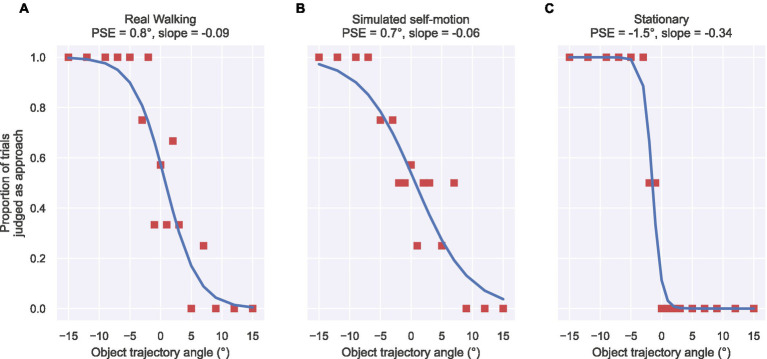
Examples of individual subject data for the Real Walking **(A)**, Simulated self-motion **(B)**, and Stationary **(C)** conditions used in psychometric analysis. Square markers depict the proportion of trials that the object was judged as approaching for each object trajectory angle (x axis). Each curve corresponds to the psychometric function (Eq. 1) fit to the subject’s data. The title above each panel shows the self-motion condition along with the fitted psychometric PSE and slope parameter values.

In Eq. 1, the independent variable θ indicates the object’s trajectory angle, the parameter p signifies the point of subjective equality (PSE) (i.e., angle at which the object trajectory was judged as neither approaching nor retreating), and the parameter s relates to the slope of the sigmoid where the function reaches its 50% point. As schematized in Figures 2B, a PSE value of 0° indicates that subjects correctly judged 0° objects as moving perpendicular to the central axis (‘cross’), a positive PSE value indicates a bias to judge trajectories as approaching (e.g., some retreating trajectories were judged as straight or approaching), and a negative PSE value indicates a bias to judge trajectories as retreating (e.g., some approaching trajectories were judged as straight or retreating). Figure 3 shows the proportion of trials judged as approaching across the trajectory angles for three representative subjects along with the respective psychometric fits. We derived the sigmoid slopes d at the PSE to estimate the discriminability (precision) of object trajectories in each self-motion condition, where d=s/4 (Gilchrist et al., 2005).

It is worth noting that this analysis provides (for each subject in each condition) a single overall estimate of the PSE and discriminability. It does not allow us to make any claims about how the accuracy or completeness of flow parsing might vary across the range of trajectory angles. However, this same limitation applies to Xie et al. (2020).

### Results and discussion

**Figure 4:**
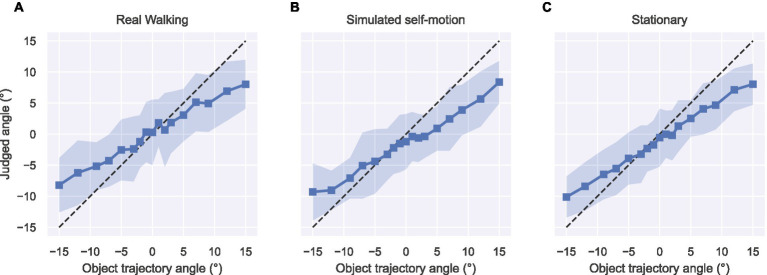
Object trajectory angle judgments in the Real Walking **(A)**, Simulated self-motion **(B)**, and Stationary **(C)** conditions of Experiment 1. Plot markers depict the mean judged angles and color bands depict the standard deviation of judgments averaged across subjects.

Figure 4 summarizes the mean object trajectory angle judgments obtained across subjects in the three self-motion conditions. To quantify the accuracy of angular judgments made by each subject, we fit each subject’s data with a straight-line function. On average, these fits account for 44–59% of the variance (mean *R*^2^), depending on the self-motion condition (Table 1). One factor that limits the reliability of the fitted slope and intercept estimates is the considerable variability of the individual subject judgments, which is reflected in the size of color bands in Figure 4 (see Supplementary Figures S1–S3 for individual subject data and straight-line model fits). By comparison, we explored whether a psychometric analysis of each subject’s data would more reliably capture the pattern of human judgments. After transforming each subject’s angular judgments into the proportion of trials each trajectory angle was judged as approaching (see Figure 3 and Methods), we fit a logistic function parameterized by the trajectory angle that coincides with its inflection point (PSE; p in Eq. 1) and a parameter related to the steepness of the sigmoidal curve (a measure of discriminability; s in Eq. 1). The logistic model yielded larger mean R^2^ values in every self-motion condition compared to the straight-line model (Table 1). In light of this improvement over the straight-line fits, we henceforth analyze the fitted psychometric PSE and discriminability parameters. This approach to analyzing the data also allows us to more directly compare our results to those of Xie et al. (2020), who had subjects judge whether the object approached or retreated.

**Table 1:** The mean proportion of variance accounted for by two regression models fit to object trajectory angle judgments in Experiment 1, as assessed by the coefficient of determination (*R*^2^).

Condition	*R*^2^ (linear model)	*R*^2^ (logistic model)
Real walking	0.439	0.625
Simulated self-motion	0.523	0.723
Stationary	0.590	0.826

Linear model refers to a straight-line fit to each subject’s angular judgments (e.g., dashed line in Figure 4). Logistic model refers to a psychometric function to each subject’s proportion approach judgments (e.g., curves in Figure 3).

**Figure 5:**
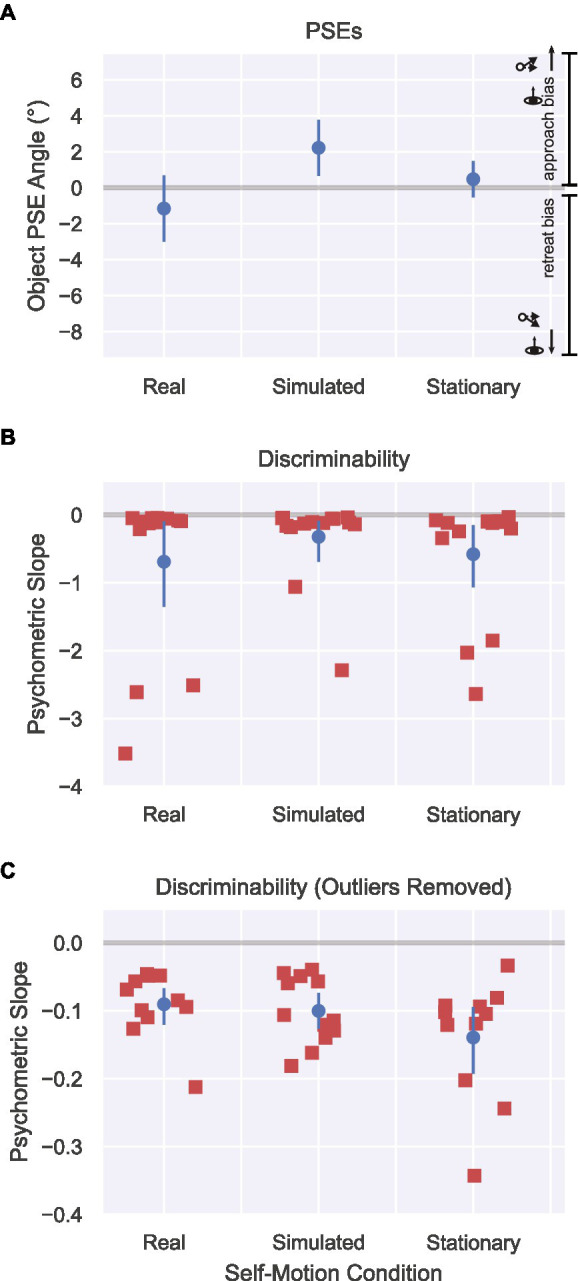
Results from Experiment 1 in the Real Walking, Simulated self-motion, and Stationary conditions. **(A)** PSEs for approach/retreat judgments determined by fitting subject responses to a psychometric curve. Positive PSEs correspond to a bias to perceive objects as approaching, while negative PSEs correspond to a bias to perceive objects as retreating. Error bars show 95% CIs. **(B)** Discriminability of subject judgments, determined according to the slope of each subject’s psychometric curve at the PSE. More negative slopes indicate better discriminability of object trajectories. Error bars show bootstrapped 95% CIs. **(C)** Same as Figure 5B but with extreme values (< −0.9) removed to better show the distribution of slopes for the other subjects.

Figure 5 shows the results of the psychometric analysis of object motion accuracy and discriminability. Let us first evaluate findings with respect to the main hypothesis that active control of locomotion may lead to greater accuracy in object perception during self-motion. A one-way repeated measures ANOVA revealed a significant effect of self-motion condition [*F*(2,26) = 11.61, *p* < 0.001, $\eta_{\text{partial}}^{2}$= 0.47]. The PSE in the Simulated self-motion condition (M = 2.21°, 95% CI [0.66°, 3.78°]) was significantly different from the Stationary condition [*t*(13) = 3.46, *p* < 0.01, Bonferroni corrected ⍺ = 0.025], indicating poorer accuracy when self-motion was simulated. It was also significantly different from zero [*t*(13) = 3.07, *p* < 0.01], indicating a bias to judge trajectories as approaching, which is consistent with the incomplete compensation of self-motion found in other studies of visual flow parsing. In other words, for subjects to perceive that the object was moving along a perpendicular path (0° trajectory), the object had to move along a retreating trajectory. Judgments in the Real Walking condition were quite different. The PSE (M = −1.16°, 95% CI [−3.01°, 0.69°]) was not significantly different than in the Stationary self-motion condition [*t*(13) = −2.08, *p* = 0.058] and not significantly different than zero [*t*(13) = −1.35, *p* = 0.20]. Although the null result does not allow us to conclude that judgments were unbiased in the Real Walking condition, it is clear that they are different than in the Simulated condition and closer to both ground truth and to judgments in the Stationary condition.

Figure 5B plots the discriminability of object motion judgments around the PSE. A one-way repeated measures ANOVA did not show an effect of self-motion condition in discriminability [*F*(2, 26) = 0.50, *p* = 0.62]. It is worth noting that in each condition, there were two or three subjects whose slopes were considerably steeper than the others, reflecting superior discriminability. Including these subjects in Figure 5B obscures the distribution of slope values for the remaining subjects. To address this issue, we replotted the data with the extreme values removed (see Figure 5C). Overall, the pattern of results is inconsistent with the results of Dokka et al. (2013), who found reduced discriminability in object motion judgments under conditions in which compensation for self-motion is greater. The results are, however, compatible with those of Xie et al. (2020) who reported no differences in discriminability between the Visual and Combined conditions.

To summarize, the pattern of results was largely consistent with that reported by Xie et al. (2020). Just as they found accurate flow parsing in their Combined condition and flow parsing gains below 1 in their Visual condition, we found judgments closer to ground truth in our Real Walking condition and an approach bias in our Simulated condition.

## Experiment 2

Experiment 2 had two aims. The first was to replicate Experiment 1, which is important because the main conclusion of that experiment – that self-motion must be actively generated for the perception of object motion to be accurate – relies partly on interpreting a non-significant difference between the mean PSE in the Real Walking condition and zero.

**Figure 6:**
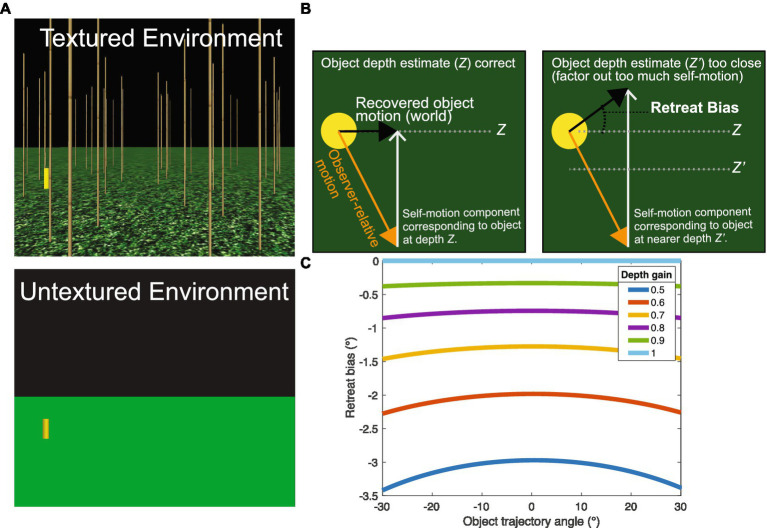
Experiment 2 overview and predictions. **(A)** Experiment 2 contained the Real Walking, Simulated, and Stationary self-motion conditions from Experiment 1, but included a condition with a textureless environment that did not generate global optic flow when the subject moved. **(B)** Predicted pattern of judgments if subjects underestimated depth in the Untextured conditions. Left: The visual system could recover the trajectory of a horizontally moving object (black arrow) from the retinal optic flow field (orange arrow) by subtracting out the self-motion component (gray arrow in left panel) appropriate for the object’s depth (Z; top dashed line in right panel). Right: Misperceiving the object depth as too close (Z’; bottom dashed line in right panel) results in the visual system factoring out too much self-motion (gray arrow in right panel) and a bias to judge the object trajectory as retreating. Black arrows indicate the hypothetical perceived trajectory of the object that matches the indicated biased judgment. **(C)** Model simulations showing bias in the recovered object trajectory (in retinal coordinates) when the depth of the object is underestimated with a gain $G_{z}<1$. Negative values indicate a bias to judge trajectories as retreating.

The second aim of Experiment 2 was to further investigate the contribution of non-visual self-motion information in accurately perceiving object motion. Whereas Experiment 1 focused on the contribution of non-visual information when visual self-motion information is also available, Experiment 2 also included conditions that allow us to assess the sufficiency of non-visual self-motion information alone. Our approach was to include conditions in which the surrounding environment was untextured (see Figure 6A, bottom panel) and hence generated no optic flow other than the local motion from the object. Also absent from the Untextured Environment were the bamboo posts, which in the Textured Environment provided both optic flow from regions above the horizon and ordinal depth information about the moving object relative to the posts. Thus, as subjects moved within the Untextured Environment, non-visual self-motion information was available but visual self-motion information was not. The untextured environment condition is similar to the non-visual condition of Xie et al. (2020), where it was found that the flow parsing gain was 0.54 (±0.4).

Unfortunately, assessing the sufficiency of non-visual information is not as simple as measuring the accuracy of judgments in the untextured environment. One complication is that humans exhibit a well-established tendency to underestimate the depth of objects in virtual environments with minimal visual structure (Loomis and Knapp, 2003; Knapp and Loomis, 2004; Armbrüster et al., 2008; Ng et al., 2016; Buck et al., 2018). Hence, removing the texture from the ground surface and the vertical posts is likely to affect not only the availability of visual self-motion information, but also the perceived depth of objects in the scene. This could, in turn, influence judgments of object motion because to properly factor out self-motion from the object’s optical motion, the visual system must take the depth of the object into account. Due to motion parallax, the component due to self-motion (i.e., the component that must be factored out) is greater when the object is nearby compared to when it is farther away. Thus, if humans are able to use non-visual information to fully compensate for self-motion, but they underestimate the depth of the object due to the impoverished environment, then the component attributed to self-motion should be greater than necessary, resulting in a bias to perceive moving objects as retreating (see Figure 6B). Of course, it is possible that humans also misperceive their self-motion, which may further complicate the interpretation of human judgments obtained from the untextured environment.

To help interpret the data from the untextured conditions, we created a mathematical model that estimates the trajectory angle of a moving object given assumptions about the accuracy of depth estimates and the (in-)completeness of flow parsing (see Appendix for details). The full version of the model has two parameters: (1) the gain with which the visual system discounts self-motion ($G_{s}$), and (2) the gain on the visual system’s estimate of object depth ($G_{z}$). To develop the reader’s intuition for how we used the model, first consider a simplified version in which *G_s_* is set to 1 by assumption and *G_z_* is allowed to vary. This assumes that the depth of the object may be misperceived but that the visual system fully factors out the component of optic flow due to self-motion at the (mis-)perceived depth. Figure 6C illustrates how the bias in perceived object trajectory would be expected to vary across different depth gains between 0.5 and 1.0. (note that the bias in Figure 6C is expressed with respect to the angle as it appears on the eye rather in the world.) Suppose that a subject misperceives an object moving along a 0° trajectory as retreating by an amount that corresponds to 2° as it appears on the eye. Inspection of Figure 6C reveals that this would be consistent with a depth gain of ~0.6.

The obvious limitation of this simplified version of the model is that it assumes that the self-motion gain is 1.0. The full version of the model addresses this shortcoming by allowing both the self-motion gain ($G_{s}$) and depth gain ($G_{z}$) to vary. Specifically, we sampled a 2D grid of values of *G_s_* and *G_z_* and for each pair counted the number of trials on which the sign of the model prediction (approach or retreat) matched the sign of the human judgment. This analysis does not reveal the single pairing of $G_{s}$ and $G_{z}$ that best captures the data but does tell us the combinations of these parameters that fit the data well. As we demonstrate below, we can use results from previous studies to further constrain the range of plausible values of *G_s_* and *G_z_*.

### Methods

The design of Experiment 2 followed that of Experiment 1 with the following exceptions.

#### Participants

Twelve subjects who were at least 18 years old and were enrolled in undergraduate psychology courses participated in the experiment and were awarded extra credit. The inclusion criteria (i.e., normal or corrected-to-normal vision) were the same as Experiment 1. The questionnaires that asked subjects to report their age were accidentally discarded so we are unable to report the mean age and age range.

#### Virtual environment

The virtual environments of the Real Walking/Textured, Simulated Self-motion/Textured and Stationary/Textured conditions in Experiment 2 were identical to the corresponding conditions in Experiment 1. In addition, subjects completed the Real Walking/Untextured and Stationary/Untextured conditions in a version of the environment that did not contain texture or bamboo posts (Figure 6A-bottom panel). We did not include a condition with simulated self-motion in the untextured environment because perceived self-motion in that condition would be indistinguishable from perceived self-motion in the Stationary/Untextured condition.

#### Procedure

Rather than verbally report the trajectory angle, subjects in Experiment 2 judged whether each object moved along an approaching or retreating trajectory. We used an adaptive staircase algorithm to draw object trajectories within ±30° of the horizontal axis on each trial and determine the PSE whereby subjects perceive trajectories as neither approaching nor retreating (0°; Kontsevich and Tyler, 1999). The order of presentation depended on the response history within each block and the algorithm evaluated the certainty associated with each PSE estimate. The algorithm selected the next trajectory angle to minimize the uncertainty of the best estimate. Each condition consisted of 40 trials, at which point the certainty of the PSE estimate always met a pre-determined level of certainty.

The depth of the object when it arrived at the central axis was a randomly selected value between 8 m and 10 m, or between 6.4 m and 8.4 m in the Stationary conditions (see Experiment 1 procedure). We adjusted the initial position accordingly so that the object arrived at the central axis after the randomly determined movement duration (2–3 s) and path length (2–4 m).

Block order was counterbalanced with three constraints. First, blocks with the same self-motion condition (e.g., real walking or stationary) were presented consecutively. Second, the Simulated Self-motion condition was never the first block of the experiment. Third, the untextured environment was never the first block of the experiment. This allowed subjects additional time to calibrate to the virtual environment.

### Results and discussion

**Figure 7:**
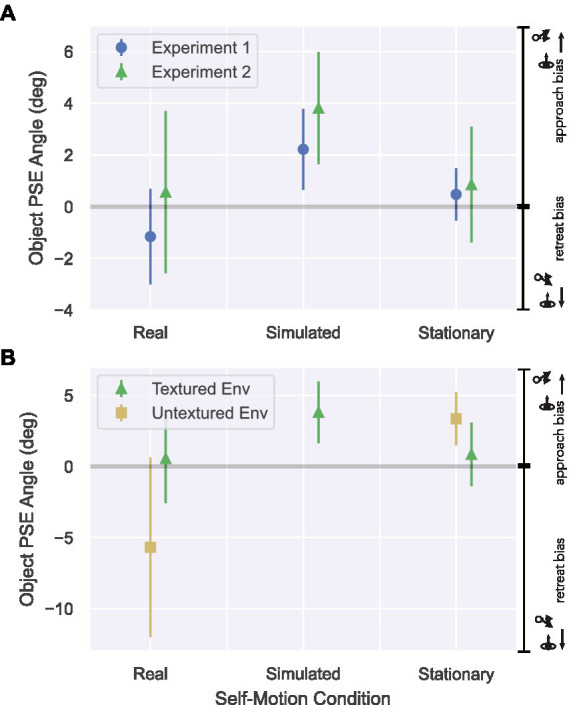
Results from Experiment 2 in the Real Walking, Simulated, and Stationary self-motion conditions. Error bars show 95% CIs. **(A)** PSE values derived from the Textured conditions in Experiment 2 (triangles) compared to those obtained in Experiment 1 (disks). Positive PSEs correspond to a bias to approach objects as approaching, while negative PSEs correspond to a bias to approach objects as retreating. **(B)** PSEs from Experiment 2 in the Textured (triangles) and Untextured (squares) environments.

Beginning with the Textured environment conditions, Figure 7A shows close agreement between the PSEs obtained in Experiments 1 and 2. Consistent with Experiment 1, a one-way repeated measures ANOVA revealed a significant effect of self-motion condition in the textured environment [*F*(2, 22) = 4.003, *p* = 0.033, $\eta_{\text{partial}}^{2}$= 0.27]. The PSE was significantly different from zero in the Simulated Self-motion condition (M = 3.81°, 95% CI [1.64°, 5.99°]) (*t*(11) = 3.86, *p* < 0.01), but not in the Real Walking (M = 0.56°, 95% CI [−2.58°, 3.71°]) (*t*(11) = 0.39, *p* = 0.70) or Stationary (M = 0.85°, 95% CI [−1.39°, 3.10°]) (*t*(11) = 0.84, *p* = 0.42) conditions. This replication of Experiment 1 reinforces the conclusion that judgments of object motion are more accurate when self-motion is real and actively generated compared to simulated.

Next, let us consider the findings from the Untextured environment conditions, in which visual self-motion information is absent and self-motion is specified by non-visual information. Recall that if the visual system completely factors out the component of the object’s optical motion due to self-motion, object trajectory judgments should exhibit a retreat bias because humans are known to underestimate depth in virtual environments with minimal visual structure (Figure 6B). The mean PSE in the Real Walking/Untextured condition (M = −5.69°) was considerably less than zero (see Figure 7B). Interestingly, the effect was not consistent across subjects, leading to more variability in this condition compared to other conditions — the 95% CI [−12.0°, 0.65°] spanned a larger range than any other condition. While a t-test showed that the PSE in the Real Walking/Untextured condition was not significantly different from zero [*t*(11) = −1.98, *p* = 0.07], it was significantly different than the PSE in the Real Walking/Textured condition [*t*(11) = −3.43, *p* = 0.006]. The PSE in the Stationary/Untextured condition was significantly different both from zero (M = 3.35°, 95% CI [1.47°, 5.24°]) [*t*(11) = −3.91, *p* = 0.002] and the PSE in the Stationary/Textured condition [*t*(11) = −2.41, *p* = 0.035].

**Figure 8:**
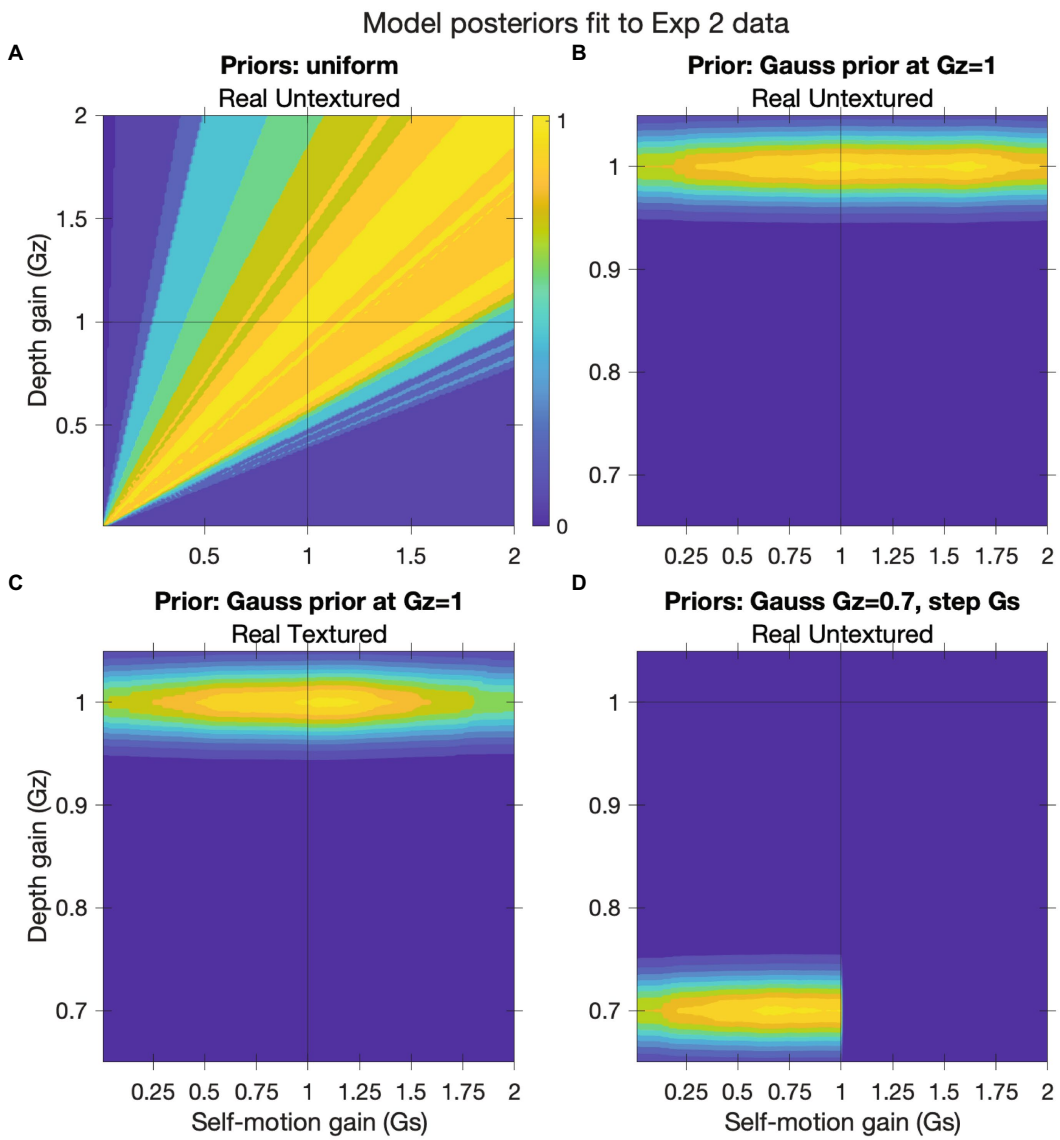
Gain analysis in Experiment 2. We fit a model to subject data that accounted for incomplete self-motion compensation (self-motion gain) and misperceptions of object depth (depth gain). **(A)** 2D histogram depicting the model fit to the human data in the Real Walking/Untextured condition with different self-motion and depth gain values. Warmer colors signify better fits between the approach/retreat prediction generated by the mathematical model (Eq. A11; see Appendix) and subject data. **(B)** Posterior distribution generated by multiplying the histogram in Figure 8A by a Gaussian prior centered at *G_z_* = 1. **(C)** Same as Figure 8B but for the Real Walking/Textured condition. **(D)** Posterior distribution generated by multiplying the histogram in Figure 8A by a Gaussian prior centered at *G_z_* = 0.7 and a step-function prior on *G_s_* with zero weight for values of *G_s_* > 1.

Recall that the rationale for including the Untextured environment in Experiment 2 was to determine whether observers could accurately estimate object motion when self-motion was specified by non-visual information alone. A retreat bias, if it exists, would be consistent with an underestimation of object depth (*G_z_* < 1) but a complete compensation for self-motion (*G_s_* = 1) at the misperceived depth. However, regardless of statistical significance, the PSE analysis on its own cannot tell us whether the degree of depth underestimation needed to account for the results is within a plausible range. In addition, there are other combinations of self-motion gain and depth gain that are also consistent with the observed judgments. The mathematical model offers a tool for estimating the combinations of gain values that could capture the data. Figure 8A shows how the fit of our mathematical model to the human data varies with self-motion gain and depth gain in the Real Walking/Untextured condition. Warmer colors indicate better fits to the human data. Thus, the best fits were found for ratios of depth gain (*G_z_*) to self-motion gain (*G_s_*) equal to or less than one; that is, when *G_z_* was equal to or less than *G_s_*.

The first key finding revealed by this analysis is that when the depth gain is assumed to be ~1.0, the only self-motion gains that allowed for good fits to the data from the Real Walking/Untextured condition were those that ranged from slightly below than 1.0 to slightly more than 1.5. This is illustrated in Figure 8B, which depicts a posterior distribution based on the product of the histogram in Figures 8A and a Gaussian prior centered at *G_z_* = 1. The bright yellow regions corresponding to the maximum a posterior (MAP) range from *G_z_* ≈ 0.9 to *G_z_* ≈ 1.6. A self-motion gain greater than 1 means that subjects perceived themselves as moving faster than they actually were, which would be incompatible with studies of human path integration in untextured environments devoid of visual self-motion information. In these studies, subjects were presented with a visual target at a given distance and then instructed to move to the location of the target with the presence or density of optic flow manipulated. Harris et al. (2002) found that when subjects attempted to reproduce the target distance in the dark, they tended to overshoot, which is consistent with a self-motion gain less than 1.0. More recently, Lakshminarasimhan et al. (2018) found that subjects overshot a previously presented visual target by a greater amount when the density of optic flow was reduced, which further supports the possibility that the self-motion gain is less than 1.0 in improvised environments. Both findings call into question the assumption that depth gain in the Real Walking/Untextured condition was ~1.0, as that assumption implies a range of self-motion gains (≥ 1.0) that is inconsistent with previous results.

Next, let us assume that the manipulation of ground texture had no effect on depth perception; that is, that the perceived depth of moving objects was the same in the Real Walking/Untextured condition and the Real Walking/Textured condition (i.e., *G_z_* = 1 in both conditions). The posterior distribution for the Real Walking/Textured condition is shown in Figure 8C. It is apparent that the most plausible values of *G_s_* in the Real Walking/Untextured condition (Figures 6B) are equal to or greater than those in the Real Walking/Textured condition (Figures 6C). In other words, if we assume that the presence or absence of ground texture had no effect on depth perception, we are led to conclude that the flow parsing gain in the Real Walking/Untextured condition was greater than or equal to the flow parsing gain in the Real Walking/Textured condition. This is inconsistent with the results of previous studies (Dokka et al., 2013; Xie et al., 2020), casting doubt on the assumption that perceived depth was the same in the two conditions. It also lends more credibility to the idea that subjects underestimated depth in the Untextured environment, which in turn contributed to the bias to perceive objects as retreating (see Figure 7B).

Lastly, we shifted the prior on depth gain to be centered on 0.7. The corresponds to an assumption that depth was underestimated by about 30% in the Real Walking/Untextured condition, which is consistent with previous studies on the perception of depth in virtual environments devoid of ground texture (see Experiment 2 introduction). We also added a prior on self-motion gain that assigns equal plausibility to values of *G_s_* less than 1 and zero plausibility to values greater than 1, reflecting an assumption that self-motion gain could not exceed 1.0 in the Real Walking/Untextured condition. The resulting posterior distribution, which is depicted in Figure 8D, indicates that the most plausible values of *G_s_* lie within the range of 0.6 to 0.9. This is slightly greater than but in the ballpark of the values reported by Xie et al. (2020).

To summarize, when the findings of Experiment 2 are interpreted in the context of previous research, they suggest that judgments in the Real Walking/Untextured condition were indeed affected by depth underestimation. We cannot draw any strong conclusions about the accuracy of flow parsing when visual self-motion information is absent. However, a plausible interpretation based on assumptions from previous studies of depth perception in untextured environments is consistent with incomplete compensation for self-motion. This is qualitatively consistent with the findings of Xie et al. (2020).

## General discussion

The primary aim of the present study was to determine whether moving human observers are capable of making accurate judgments of the trajectory of a moving object when self-motion is real and actively generated rather than simulated or passive. In two experiments, we found that when subjects moved through a densely textured virtual environment under their own control, perceived trajectory angle was not significantly different from actual trajectory angle. In contrast, when self-motion was simulated, subjects exhibited a bias to perceive objects as moving along an approaching trajectory. This is consistent with a tendency to discount less than 100% of the optic flow due to self-motion when self-motion is specified by visual information alone. Taken together, the results suggest that non-visual information generated during real, actively generated self-motion is necessary to make accurate judgments of object motion.

While non-visual information is necessary, there is a lack of evidence that such information is sufficient. The results of Experiment 2, when taken together with findings from other studies on depth perception in untextured environments, suggest that subjects factored out less than 100% of the local flow due to self-motion when global optic flow was absent. We emphasize that this interpretation relies on the assumption that depth was underestimated to a larger degree in the untextured environment. However, this assumption is grounded in previous research on depth perception. Furthermore, if we assume no differences in perceived depth across textured and untextured environments, it leads to the unlikely conclusion that flow parsing gain was greater in the untextured environment. This lends credibility to the assumption that underlies the analysis and conclusions of Experiment 2.

Our findings are consistent with those of Xie et al. (2020) in that subjects made accurate judgments of world-relative object motion while walking in a visually rich environment but failed to completely compensate for self-motion when it was simulated. The findings from our Untextured environment in Experiment 2 are also compatible with those of their Non-visual condition, where the flow parsing gain was less than 1.0. The novel contribution of the present study is that the conditions used in our study were more similar to those encountered during real-world locomotor tasks. The fact that the findings of the two studies were similar helps to alleviate possible concerns that the conclusions of Xie et al. might have been restricted to the unnatural viewing conditions that were tested.

The present study also makes a methodological contribution by introducing a novel approach for interpreting data that allows the researcher to use results from previous studies to constrain the range of plausible parameters. In Experiment 2, we demonstrated how this approach can be useful to estimate the ranges of self-motion gains and depth gains that are compatible with both the new data and previous findings. This approach could prove useful in future studies in which biases in depth perception (such as those that are known to exist in virtual environments) may affect perceptual judgments of other properties.

There is one sense in which our findings differ from those of Xie et al. (2020). Their approach to addressing the concern about depth misperception in textureless environments was to conduct a control experiment designed to compare judgments of distance to a probe resting on a solid, uniform ground plane versus a random-dot ground plane. They found no significant difference in distance judgments across the two conditions and concluded that depth perception was consistent regardless of the appearance of the ground surface (solid or random-dot). For this to be the case in our study, the self-motion gain in the Real Walking/Untextured condition of Experiment 2 would have to have been greater than or equal to the self-motion gain in the Real Walking/Textured condition. We can think of no reason why that would be the case, leading us to conclude that subjects must have underestimated depth to a larger degree in the Real Walking/Untextured condition of our study.

How do we reconcile this finding with that of the control experiment in Xie et al. (2020)? One possibility is that their random-dot and empty ground environments were more similar in terms of amount of visual structure than our Textured and Untextured environments. In their study, the two environments differed only in that one included white dots that were sparsely distributed on a ground surface. In our study, the Textured environment comprised a densely textured ground plane with vertical posts (see Figure 6A), which provided salient static and dynamic depth information.

Our results also suggest that object motion perception during locomotion is not only accurate, but precise. This may seem somewhat surprising given evidence from a previous study (Dokka et al., 2013) showing poor discriminability when self-motion was real compared to when it was simulated. However, the findings are not contradictory, as self-motion was passive in Dokka et al. and actively generated in the present study. Indeed, Xie et al. (2020) also found that discriminability in the Combined condition was comparable to that in the Visual condition. Together, the findings that discriminability is at least as good when self-motion was actively generated compared to when it is simulated is not compatible with the explanation offered by Dokka et al. They attributed the drop-off in discriminability to an increase in noise that is assumed to occur when information from multiple sensory arrays is available. If this explanation is correct, we would have expected discriminability to be worse in the Real Walking condition compared to the Simulated Self-motion condition of the present study. It is possible that noise is actually reduced rather than amplified when self-motion is actively generated rather than passive as the visual system is also well calibrated to anticipate the pattern of motion generated by locomotion over the ground.

Although the present study and Xie et al. (2020) are the first two studies to measure the accuracy of object motion perception during real, actively generated locomotion, Dupin and Wexler investigated object motion perception during head movements, another form of actively generated self-motion (Dupin and Wexler, 2013). Subjects judged the clockwise or counterclockwise rotation of a plane after performing head movements or while remaining stationary. Consistent with findings from the visual flow parsing literature and those from our Simulated condition, Dupin and Wexler found that when self-motion was simulated through the rotation of a background plane, subjects only factored out 37% (on average) of self-motion. Self-motion compensation improved to 75% when head movements were real and actively generated, but it did not reach the accurate judgments of subjects in the present experiment. This discrepancy may arise for several reasons. First, subjects may have found the task of judging the direction of rotation of a plane more difficult than judging the trajectory angle of an object translating on a ground plane. In our Stationary condition, subjects judged the object trajectory with nearly veridical accuracy, but there is no indication in the Dupin and Wexler study that subjects correctly judged the planar rotation in any circumstance, with or without movement of the background. Second, the visual system may be calibrated to accurately judge object motion during locomotion, due to the frequency at which humans encounter moving objects while walking. Active head movements alone may not generate comparably strong non-visual self-motion signals to accurately recover world-relative object motion.

In summary, the findings of the present study support the conclusions of Xie et al. (2020) that humans can make accurate and precise judgments of object motion during locomotion. Our study goes beyond Xie et al. (2020) by demonstrating accurate flow parsing under conditions that are more similar to those encountered during ecologically valid locomotor tasks. Taken together, these studies demonstrate that the information available when self-motion is real and actively generated plays an important role in the perception of object motion in world coordinates.

## Data availability statement

The code to simulate the mathematical model is available at https://github.com/owlayton/MM-Flow-Parsing-Gain_Release.

## Ethics statement

The studies involving human participants were reviewed and approved by Institutional Review Board at Rensselaer Polytechnic Institute. The patients/participants provided their written informed consent to participate in this study.

## Author contributions

OL: methodology, software, validation, formal analysis, data curation, writing – original draft, writing – review and editing, visualization. MP: conceptualization, methodology, software, formal analysis, investigation, data curation, writing – original draft, visualization. BF: conceptualization, methodology, software, formal analysis, resources, writing – review and editing, supervision, project administration, funding acquisition, visualization. All authors contributed to the article and approved the submitted version.

## Funding

This work was supported by grants from the Office of Naval Research (N00014-14-1-0359) and the National Institutes of Health (1R01EY019317). The funders had no role in study design, data collection and analysis, decision to publish, or preparation of the manuscript.

## Conflict of interest

The authors declare that the research was conducted in the absence of any commercial or financial relationships that could be construed as a potential conflict of interest.

## Publisher’s note

All claims expressed in this article are solely those of the authors and do not necessarily represent those of their affiliated organizations, or those of the publisher, the editors and the reviewers. Any product that may be evaluated in this article, or claim that may be made by its manufacturer, is not guaranteed or endorsed by the publisher.
