# Supplementary material for: The accuracy of object motion perception during locomotion
Source: Front Psychol. 2023 Jan 12;13:1068454. doi: 10.3389/fpsyg.2022.1068454 (PMC9878598; doi:10.3389/fpsyg.2022.1068454)
Supplement: Supplementary file 1 [file Data_Sheet_1.pdf]

frontiers

Supplementary Material

The Accuracy of Object Motion Perception during Locomotion

Oliver W. Layton*, Melissa S. Parade and Brett R. Fajen

*** Correspondence:** Corresponding Author: oliver.layton@colby.edu

1 Appendix: Mathematical model of object motion estimation during self-motion

We developed a mathematical model of human object motion judgments that considers the possibility that subjects may have both misperceived object depth and attributed more or less optic flow to self-motion. Figure 6c illustrates model predictions about how an underestimation of depth could influence judgments of the moving object trajectory. We used the model to examine the plausibility that different degrees of depth and/or self-motion misperceptions could account for human judgments in Experiment 2 (Figure 8). We implemented the model using MATLAB R2022a and the code is available at: https://github.com/owlayton/MM-Flow-Parsing-Gain_Release.

Our analysis begins by considering the optic flow on the retina of a moving observer. We assume a standard 3D coordinate system (Raudies & Neumann, 2013) in which the eye of the observer with height $h = 1.7m$ is initially positioned at $\vec{P} = \left(P_x, P_y, P_z\right) = (0, h, 0)$. The positive Z axis aligns with the straight-ahead direction of observer translation (0° heading), the positive Y axis corresponds to the upward direction above a hypothetical ground plane ($Y = 0$), and the positive X direction corresponds to the rightward direction relative to the observer facing the positive Z axis. We use a standard pinhole camera model to project the point (X, Y, Z) in the world onto corresponding coordinates (x, y) of a 2D planar surface representing the observer's retina:

$$(x, y, f) = \frac{f}{Z - P_z}\left[(X, Y, Z) - \vec{P}\right] = \frac{f}{Z - P_z}\left(X - P_x, Y - P_y, Z - P_z\right) = \frac{f}{Z_r}(X_r, Y_r, Z_r) \quad \text{Eq. A1.}$$

In Eq. A1, f represents the focal length (cm) of the model eye and (X_r, Y_r, Z_r) represents the relative position between the observer and point in the world. We carry f through our calculations but assume $f = 1\ cm$ in our simulations.

We define observer movement through the world according to the translation vector $\vec{T_s} = \left(T_{x_s}, T_{y_s}, T_{z_s}\right)$. Since subjects in the main experiments walked in approximately straight forward paths, we assume that the model observer only translated along the positive Z axis. That is, $T_{x_s} = T_{y_s} = 0$ and T_{z_s} corresponds to the speed of self-motion, which we set to 0.8 m/s to match the mean walking speed in Experiment 1. In a stationary environment without a moving object, the optic flow experienced by the observer is specified by (Longuet-Higgins & Prazdny, 1980):

$$\vec{O_s} = \frac{1}{Z_r}\begin{pmatrix} -f & 0 & x \\ 0 & -f & y \end{pmatrix} \cdot \begin{pmatrix} 0 \\ 0 \\ T_{z_s} \end{pmatrix} = \frac{1}{Z_r}\left(xT_{z_s}, yT_{z_s}\right) \qquad \text{Eq. A2.}$$

Now we consider the presence of an object that moves independently of the observer with translation vector $\vec{T_t} = \left(T_{x_t}, T_{y_t}, T_{z_t}\right)$. Because in the experiments the object only moved laterally toward the locomotor axis and possibly in depth, $T_{y_t} = 0$. If the object moves at the same 2.5 m/s speed as in the experiments, the lateral $\left(T_{x_t}\right)$ and depth $\left(T_{z_t}\right)$ velocity components of the object moving along angle θ are:

$$T_{x_t} = 2.5 \ \cos\theta \qquad \text{Eq. A3.}$$
$$T_{z_t} = 2.5 \ \sin\theta \qquad \text{Eq. A4.}$$

The net relative translation $(\overrightarrow{T_r})$ between the observer and object is given by

$$\overrightarrow{T_r} = \overrightarrow{T_s} - \overrightarrow{T_t} \qquad \text{Eq. A5.}$$

and the resultant optic flow is specified by:

$$\vec{O_r} = \frac{1}{Z_r}\begin{pmatrix} -f & 0 & x \\ 0 & -f & y \end{pmatrix} \cdot \begin{pmatrix} -T_{x_t} \\ 0 \\ T_{z_s} - T_{z_t} \end{pmatrix} = \frac{1}{Z_r}\left(fT_{x_t}, yT_{z_r}\right) \qquad \text{Eq. A6.}$$

In Eq. A6, T_{z_r} represents the relative translation in depth ($T_{z_r} = T_{z_s} - T_{z_t}$) by Eq. A5 and Z_r represents the relative depth. For the expression on the right-hand side, we set $x = 0$ consistent with the fact that subjects judged the object trajectory at the end of trial when the object intersected the locomotor axis. We carry y through our calculations but in simulations we fix the parameter to a constant value that corresponds to the top of the moving object. By Eq. A1, $y = {}^{(0.5m - 1.7m)}\!/_{7.4\ m} = -0.16$, where we assume $Z_r = 7.4m$, the average final relative depth in Experiment 2, and the object has a height of 0.5 m.

The following equation models the flow parsing process whereby the visual system recovers an estimate of the optic flow corresponding to the object $\widehat{\overrightarrow{O_t}}$ from the retinal flow $\overrightarrow{O_r}$ and the estimated optic flow component due to the observer $\widehat{\overrightarrow{O_s}}$.

$$\widehat{\overrightarrow{O_t}} = \overrightarrow{O_r} - \widehat{\overrightarrow{O_s}} \qquad \text{Eq. A7.}$$

We account for possible misperceptions of object depth Z_r and self-motion speed T_{z_s} that influence the self-motion component $\widehat{\overrightarrow{O_s}}$ by introducing multiplicative depth (G_z) and self-motion (G_s) gains. For example, $G_z = 0.8$ would correspond to a 20% underestimation of depth, $G_z = 1$ would correspond to an accurate depth estimate, and $G_z = 1.2$ would correspond to a 20% overestimation of depth. This approach adopts the simplifying assumption that perceived depth is linearly related to actual depth

(Loomis & Philbeck, 2008). After plugging in Eqs. A2 and A6 into Eq. A7 and introducing the gains, we obtain:

$$\widehat{\overrightarrow{O_t}} = \frac{1}{Z_r}\left(fT_{x_t}, yT_{z_r}\right) - \frac{1}{G_z Z_r}\left(0, yG_s T_{z_s}\right) = \frac{1}{Z_r}\left(fT_{x_t}, y\left(T_{z_r} - \frac{G_s}{G_z}T_{z_s}\right)\right) \qquad \text{Eq. A8.}$$

We recover the estimated trajectory angle of the object $\hat{\theta}$ on the projection plane according to:

$$\hat{\theta} = \tan^{-1}\left(\frac{\widehat{O_{t_y}}}{\widehat{O_{t_x}}}\right) = \tan^{-1}\left(\frac{y\left(T_{z_r} - \frac{G_s}{G_z}T_{z_s}\right)}{fT_{x_t}}\right) \qquad \text{Eq. A9.}$$

1.1 Influence of depth gain

In the simulations corresponding to Figure 6c that focus on the influence of depth gain, we fixed $G_s = 1$ and sampled object trajectory angles $\theta \in [-30°, 30°]$ and $G_z \in [0.5, 1]$ in steps of 0.1. We plotted the bias $\widehat{\theta_b}$ in the estimated trajectory angle on the projection plane, defined by the difference in the angle recovered using Eq. A9 when the true self-motion component is factored out ($\theta_o; G_z = 1$) and when the sampled G_z value is used (θ_{G_z}):

$$\widehat{\theta_b} = \theta_o - \theta_{G_z} \qquad \text{Eq. A10.}$$

1.2 Bayesian analysis of Experiment 2

In the simulations corresponding to Figure 8, we used the mathematical model to determine the plausibility that certain combinations of self-motion and depth gains could account for each subject's approach/retreat judgments in Experiment 2. For each combination of gain values sampled between 0 and 2 ($G_z, G_s \in (0, 2]$) in steps of 0.01, we derived an object trajectory angle estimate using Eq. A9. For the ith gain combination$\left(G_{z_i}, G_{s_i}\right)$, subject s, and self-motion/environment condition c, we tallied the number of trials (≤40) for which the sign of the model estimate matched the approach (-1) or retreat (+1) human judgment (see Supplementary Figure S4):

$$H_{c,s,G_{z_i},G_{s_i}} = \sum_{trial\ j}\left(sign\left(\hat{\theta}_{c,s,G_{z_i},G_{s_i},j}\right) == R_{o\,c,s,G_{z_i},G_{s_i},j}\right) \qquad \text{Eq. A11.}$$

In Eq. A11, $==$ denotes the equality operator, $\hat{\theta}_{c,s,G_{z_i},G_{s_i},j}$ indicates the model estimate (Eq. A9), and $R_{o\,c,s,G_{z_i},G_{s_i},j}$ indicates the human judgment, both on trial j. As Supplementary Figure S4 depicts, $H_{c,s,G_{z_i},G_{s_i}}$ can be thought of as a 2D histogram for each subject and condition where each entry indicates the number of trials for which the sign of model estimate and the human judgment agree.

To quantify the probability that each pair of gain values accounts for human judgments, we computed a posterior distribution for each condition c. To do this, we summed the histograms across subjects and applied a prior $F(G_z, G_s)$ on the G_z values, G_s, values, or both:

$$P_{c,G_{z_i},G_{s_i}} = \sum_{s=1}^{12} H_{c,s,G_{z_i},G_{s_i}} F(G_z, G_s) \qquad \text{Eq. A12.}$$

Figure 8 shows the posterior distribution for the Real Walking/Untextured and Real Walking/Textured conditions with difference choices of priors summarized in Supplementary Table S2.

2 References

Loomis, J. M., & Philbeck, J. W. (2008). Measuring spatial perception with spatial updating and action. In *Embodiment, ego-space, and action* (pp. 17-60). Psychology Press.

Longuet-Higgins, H. C., & Prazdny, K. (1980). The interpretation of a moving retinal image. *Proceedings of the Royal Society of London. Series B. Biological Sciences*, *208*(1173), 385-397. Retrieved from https://royalsocietypublishing.org/doi/abs/10.1098/rspb.1980.0057

Raudies, F. & Neumann, H. (2013). Modeling heading and path perception from optic flow in the case of independently moving objects. *Front Behav Neurosci*, *7*, 23. https://doi.org/10.3389/fnbeh.2013.00023

3 Supplementary Figures and Tables

3.1 Supplementary Figures

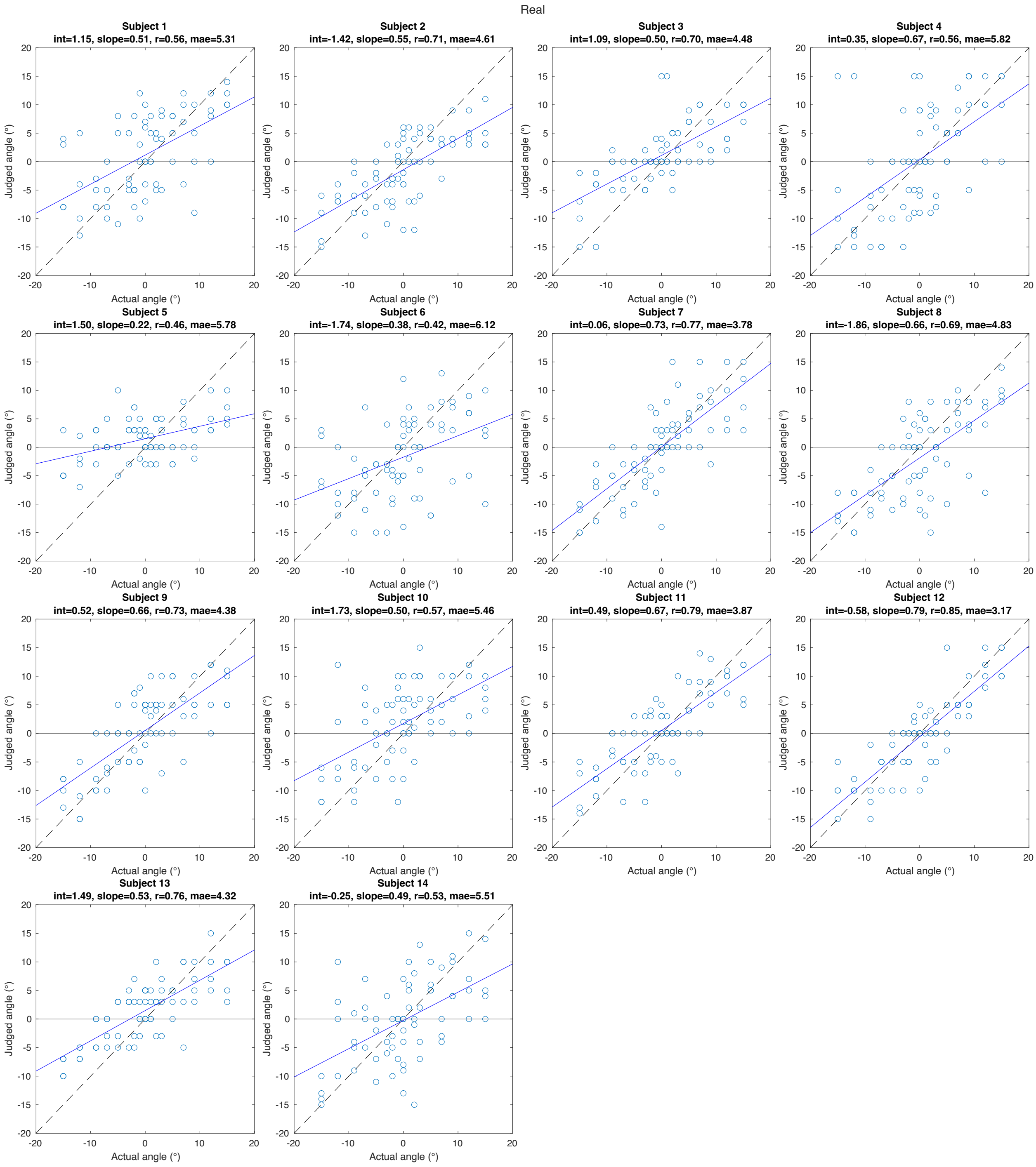

Supplementary Figure S1. Object trajectory angle judgments of individual subjects from the Real Walking condition in Experiment 1. Blue curve depicts the linear regression model fit. Dashed curve depicts the unity line. The title above each panel includes the intercept (int) and slope that specifies each fitted linear model, as well as the correlation coefficient (r) and the mean absolute error (mae).

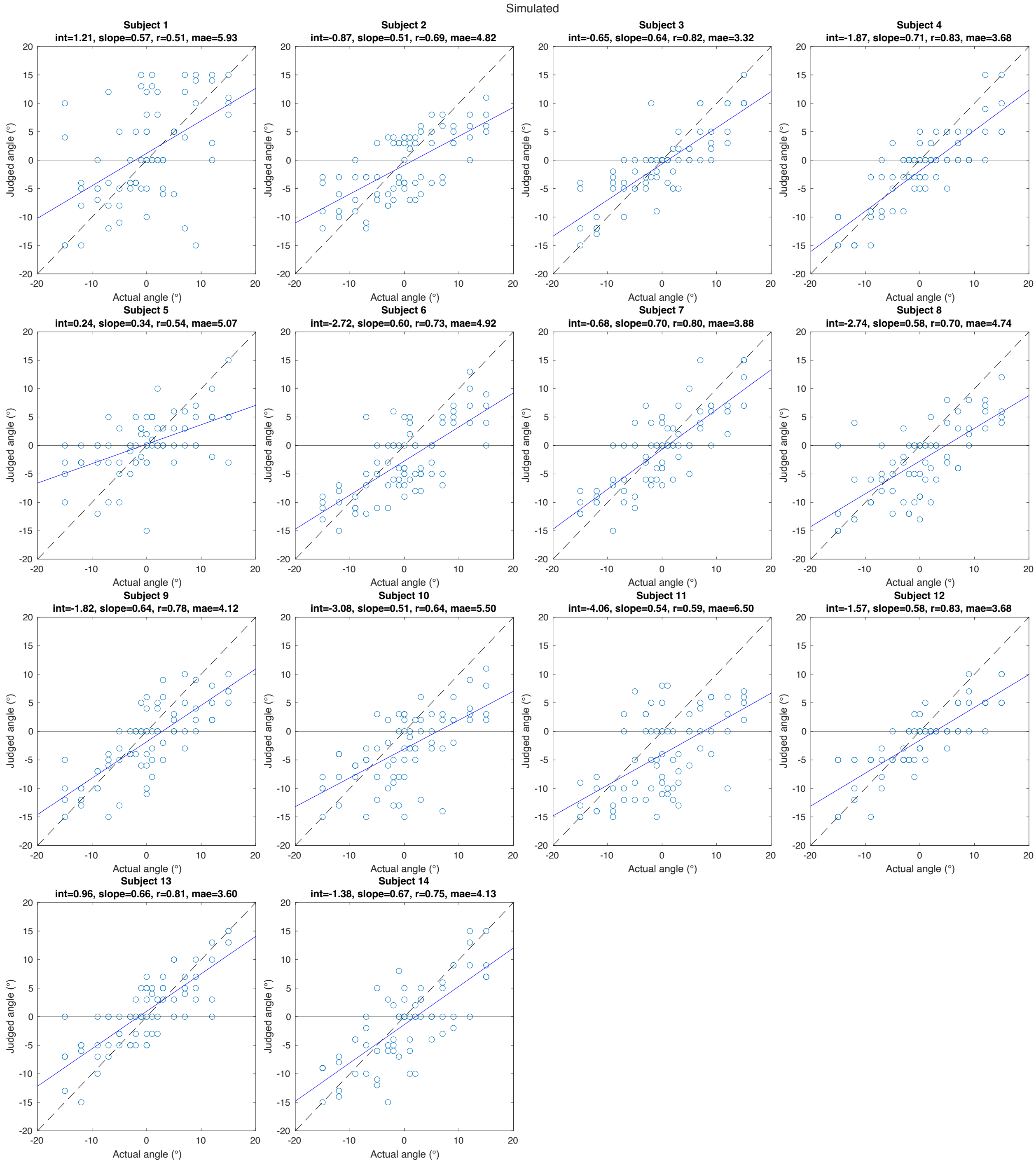

Supplementary Figure S2. Object trajectory angle judgments of individual subjects from the Simulated self-motion condition in Experiment 1. Same format as Supplementary Figure S1.

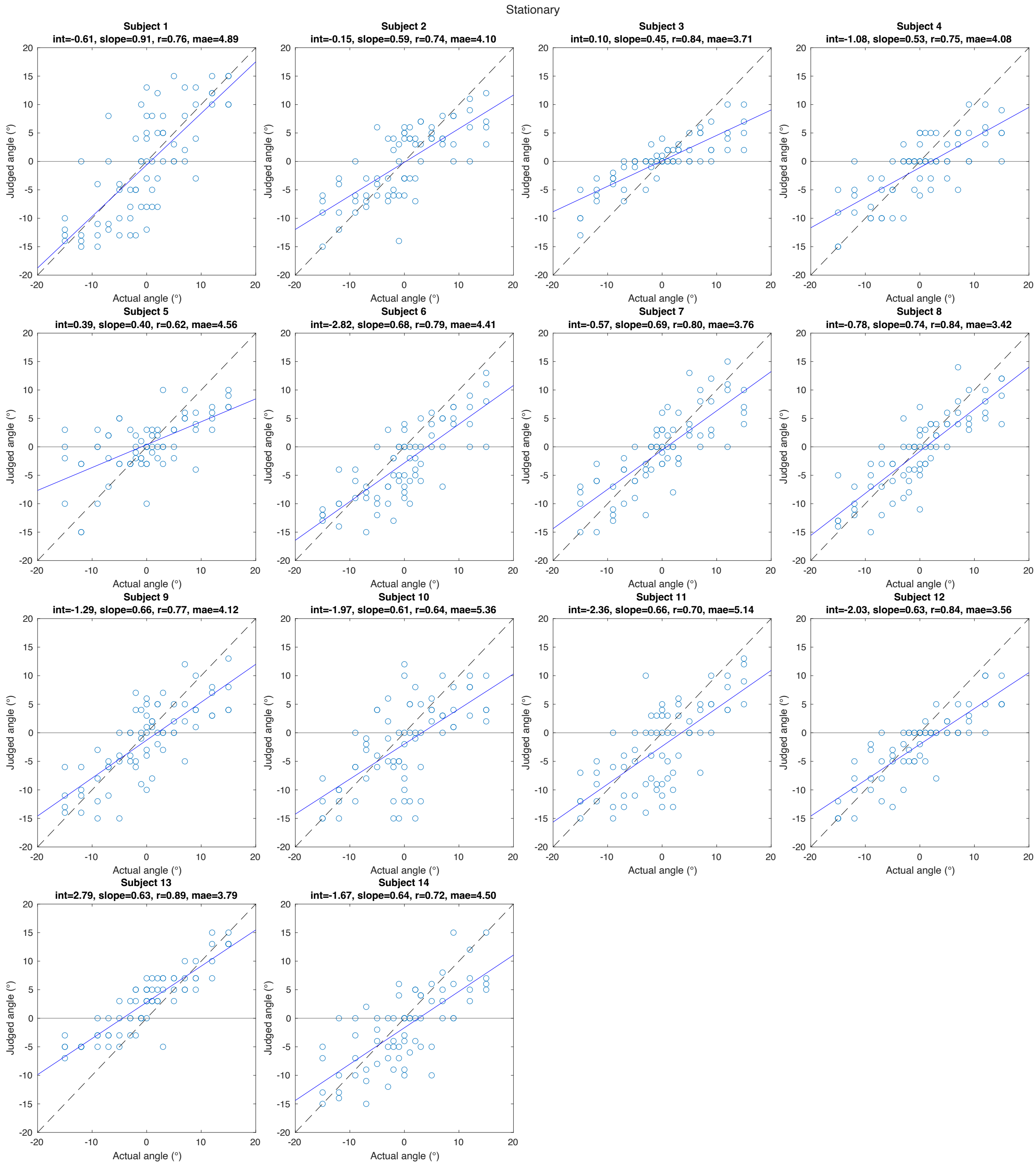

Supplementary Figure S3. Object trajectory angle judgments of individual subjects from the Stationary condition in Experiment 1. Same format as Supplementary Figure S1.

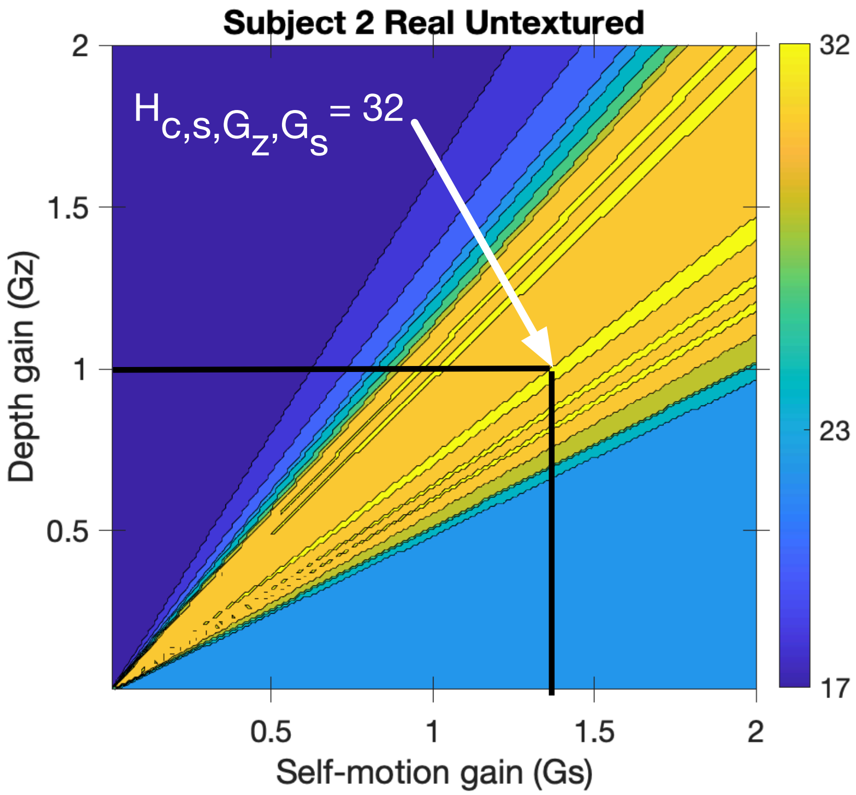

Supplementary Figure S4. Histogram of sign matches over 40 trials between the model and judgments of Subject 2 in the Real Walking/Untextured condition. The arrow points to a combination of self-motion and depth gains that results in model approach/retreat estimates that are consistent with Subject 2 on 32/40 trials of the Real Walking/Untextured condition.

3.2 Supplementary Tables

	**Real Walking condition**	**Simulated self-motion condition**	**Stationary condition**
**Intercept (M ± CI)**	0.18° ± 0.63°	-1.36° ± 0.80°	-0.86° ± 0.74°
**Slope (M ± CI)**	0.56 ± 0.08	0.59 ± 0.05	0.63 ± 0.06
**Correlation coefficient (M ± CI)**	0.65 ± 0.07	0.71 ± 0.06	0.77 ± 0.04
**Mean absolute error (M ± CI)**	4.82° ± 0.46°	4.56° ± 0.50°	4.24° ± 0.31°

Supplementary Table S1. Statistics describing the accuracy of object trajectory judgments and linear models fit to these data from Experiment 1. Each cell contains the mean and 95% confidence interval computed across 14 subjects in the indicated condition.

**Figure**	$\boldsymbol{G_z}$ **Prior**	$\boldsymbol{G_s}$ **Prior**
8a	U(0, 2)	U(0, 2)
8b	N(1, 0.025^2)	U(0, 2)
8c	N(1, 0.025^2)	U(0, 2)
8d	N(0.7, 0.025^2)	U(0, 1); 0 for $G_s > 1$

Supplementary Table S2. Priors applied to counts corresponding to depth (G_z) and self—motion (G_s) gain parameters. $U(x, y)$ indicates a uniform distribution between the end points x and y. $N(\mu, \sigma^2)$ indicates the normal distribution centered at μ with variance σ^2.
